# SARS and Common Viral Infections

**DOI:** 10.3201/eid1006.030863

**Published:** 2004-06

**Authors:** Janice K. Louie, Jill K. Hacker, Jennifer Mark, Shilpa S. Gavali, Shigeo Yagi, Alex Espinosa, David P. Schnurr, Cynthia K. Cossen, Erin R. Isaacson, Carol A. Glaser, Marc Fischer, Arthur L. Reingold, Duc J. Vugia, for the Unexplained Deaths, Critical Illnesses Working Group

**Affiliations:** * California Emerging Infections Program, Berkeley, California, USA;; † California Department of Health Services, Berkeley, California, USA;; ‡ Centers for Disease Control and Prevention, Atlanta, Georgia, USA

**Keywords:** severe acute respiratory syndrome, SARS, polymerase chain reaction, molecular testing

## Abstract

In California, molecular testing was useful in decreasing suspicion for severe acute respiratory syndrome (SARS), by detecting common respiratory pathogens (influenza A/B, human metapneumovirus, picornavirus, *Mycoplasma pneumoniae*, *Chlamydia* spp., parainfluenza virus, respiratory syncytial virus, and adenovirus) in 23 (45%) of 51 patients with suspected SARS and 9 (47%) of 19 patients with probable SARS.

Severe acute respiratory syndrome (SARS) has become the new paradigm for the global havoc that can be produced by an emerging infectious disease (*1*). As of July 31, 2003, a total of 8,096 probable SARS cases had been reported to the World Health Organization from 29 countries or areas, with 774 deaths and a case-fatality ratio of 9.6% (*2*). California was particularly affected by the SARS outbreak, reporting one-fifth of suspect or probable cases in the United States (15% of suspect SARS cases and 26% of probable SARS cases), with two serologically confirmed cases. In 2002, the California Unexplained Pneumonia (CUP) Project, a respiratory surveillance project that uses enhanced laboratory techniques to identify etiologic agents of severe pneumonia, was initiated at the California Department of Health Services (CDHS) in collaboration with the Centers for Disease Control and Prevention (CDC) Emerging Infections Program. The CUP project’s extensive diagnostic testing algorithm was applied to specimens submitted to CDHS for SARS testing.

## The Study

From March 12, 2003, through July 30, 2003, cases of possible SARS reported to the CDHS were classified as suspect, probable, or laboratory-confirmed, according to CDC criteria (*3*). Extensive diagnostic testing was performed at the CDHS Viral and Rickettsial Disease Laboratory on specimens from 165 patients, including those with conditions that did not meet strict CDC case criteria (Table 1).

**Table 1:** Respiratory testing algorithm^a^

Respiratory specimens	Serologic testing^b^
Viral culture	Acute-phase serum specimens: IgM
Polymerase chain reaction	*Chlamydia* spp.
Influenza A	*Mycoplasma pneumoniae*
Influenza B	Paired serum specimens: IgG
Respiratory syncytial virus	*Chlamydia* spp.
Parainfluenza virus types 1–4	*M. pneumoniae*
Human metapneumovirus	Influenza A and B
Coronavirus OC43 and 229E	Respiratory syncytial virus
Adenovirus	Parainfluenza virus types 2–4
Picornavirus	Adenovirus
SARS-CoV	SARS-CoV

^a^Ig, immunoglobulin; SARS-CoV, severe acute respiratory syndrome–associated coronavirus. ^b^All serologic assays were in-house enzyme immunoassays (*4*), except for the Meridian IgM assay for *M. pneumoniae* (Meridian Bioscience, Inc., Cincinnati, OH) and enzyme-linked immunosorbent assay of the Centers for Disease Control and Prevention for SARS-CoV.

Submitted specimens were transported on cold pack and either frozen at –70°C or processed immediately. A total of 281 respiratory specimens and 78 serum specimens were analyzed, including 210 nasopharyngeal swabs, 23 nasal swabs, 17 throat swabs, 15 nasal washes, 11 sputum specimens, 5 endotracheal aspirates, 39 single acute-phase serum specimens, and 39 acute- and convalescent-phase paired serum specimens. Convalescent-phase serum specimens were collected at least 28 days after symptom onset. Because of difficulties obtaining convalescent-phase sera, specimens from only 32 case-patients underwent combined testing by polymerase chain reaction (PCR), culture, and serologic methods.

Total nucleic acid was extracted from all respiratory specimens for reverse transcriptase (RT)-PCR by using the MasterPure Complete DNA and RNA Purification Kit (Epicentre Technologies, Madison, WI). RT-PCR assays were performed according to Erdman et al. (*5*) with primers for respiratory syncytial virus (RSV), parainfluenza virus (PIV) types 1–3, and influenza A and B; PIV 4 (*6*); coronavirus (CoV) 229E reverse primer (*7*), and human metapneumovirus (HMPV) (*8*). Primers for CoV OC43 and CoV 229E forward primer were modified from Myint et al. (*9*). We used adenovirus and picornavirus primers (adenovirus: forward primer 5´ - CCC(AC)TT(CT)AACCACCACCG – 3´ and reverse 5´ - ACATCCTT(GCT)C(GT)GAAGTTCCA – 3´; picornavirus: forward primer 5´ - GGCCCCTGAATG(CT)GGCTAA - 3´ and reverse 5´ - GAAACACGGACACCCAAAGTA - 3´). Reaction products were visualized on ethidium bromide–stained agarose gels with ultraviolet illumination. RT-PCR for SARS-CoV was performed on an iCycler (BioRad Laboratories, Inc., Hercules, CA) by using the TaqMan One-step RT-PCR Master Mix (Applied Biosystems, Foster City, CA) with primers and probes developed at CDC.

Based on β-actin gene amplification, specimens from 151 patients were suitable for molecular testing. In 63 (42%) of these, RT-PCR detected a respiratory pathogen (Table 2). No patient had more than one agent identified by molecular methods. In addition, respiratory samples with adequate volume were added to Vero E6, primary rhesus monkey kidney cells, and human fetal diploid lung cells, according to standard diagnostic procedures. Viruses were isolated from 16 (10%) of 154 patients (Table 2). All specimens yielding positive viral cultures were also positive by RT-PCR. Overall, RT-PCR assays were more sensitive than culture; for example, of the 26 patients who had influenza A detected by RT-PCR, 9 were culture-positive.

**Table 2:** Detection of respiratory pathogens by polymerase chain reaction (PCR), culture, and serologic testing for cases tested at the California Department of Health Services^a^

	PCR (N = 151) n (%)	Culture (N = 154) n (%)	Serologic testing^b^ (N = 78) n (%)
Influenza A	27 (18)^c^	9 (6)	4 (5)^d^
Influenza B	1 (1)	1 (1)	1 (1)^d^
Respiratory syncytial virus	5 (3)	1 (1)	1 (1)^d^
Parainfluenza virus types 2–4	6 (5)	5 (4)	0
Human metapneumovirus	11 (7)	0	ND
Coronavirus OC43	1 (1)	0	ND
Coronavirus 229E	0	0	ND
Parainfluenza virus type 1	0	0	ND
Adenovirus	0	0	1 (1)^d^
Picornavirus	12 (8)	0	ND
Mycoplasma pneumoniae	0^e^	ND	3 (4)^c^
*Chlamydia* spp.	0^e^	ND	1 (1)
SARS-CoV	0	0	2 (3)
Total positive	63 (42)	16 (10)	13 (17)

^a^ND, not done; SARS-CoV, severe acute respiratory syndrome–associated coronavirus. ^b^Measured as a significant rise in immunoglobulin (Ig) G in paired serum samples for all specimens except one positive for *M. pneumoniae* IgM. ^c^Specimens from one case-patient positive for influenza A (by PCR and culture) were also positive for *M. pneumoniae* IgM. ^d^Specimens from one case-patient negative by culture and PCR. ^e^*Mycoplasma pneumoniae* and *Chlamydia pneumoniae* PCRs were performed retrospectively only on specimens from patients with serologic evidence of *M. pneumoniae* (n = 3) and *Chlamydia* spp. (n = 1) infection.

Serologic testing was performed on specimens from 78 patients (Table 2). A significant rise in immunoglobulin (Ig) G was seen in 10 of the 39 patients who had paired acute- and convalescent-phase serum specimens (*M. pneumoniae* [2 patients], influenza A [4 patients], 1 each of *Chlamydia* spp., RSV, influenza B, and adenovirus). Of the 39 patients from whom a single serum sample was collected, two patients had detectable IgM (one each of *Chlamydia* spp. and *M. pneumoniae*). The patient with *M. pneumoniae* also had influenza A detected by PCR and culture; this patient was the only one with evidence of possible co-infection. Seven putative causal agents were identified by serologic testing alone without corresponding positive findings by PCR or culture, including one each of influenza A, influenza B, RSV, and adenovirus. PCR assays performed retrospectively on specimens from patients with positive serologic results for *M. pneumoniae* and *Chlamydia* spp. were negative for those organisms. Specimens from an additional two patients showed rises in IgG to multiple antigens, consistent with a nonspecific immune response. No respiratory specimens were positive for SARS-CoV by RT-PCR, although serologic tests of samples from two patients were positive for SARS-CoV antibody.

Sequence analyses confirmed the identity of HMPV and influenza A RT-PCR amplification products. A BLAST (available from: www.ncbi.nih.gov/BLAST) comparison of five putative HMPV specimens showed identity to HMPV sequences in GenBank. Similarly, influenza A amplification products from 12 patients identified solely by RT-PCR showed homology to known influenza A viruses.

Of the 165 patients tested, 51 (31%) met the criteria for suspect SARS, 19 (12%) met the criteria for probable SARS, and 2 had serologically confirmed SARS (Table 3). A likely pathogen was detected in 23 (45%) of the 51 suspect and 9 (47%) of the 19 probable SARS patients (Table 3). RT-PCR was the most sensitive diagnostic method, identifying a likely causal agent in 21 (41%) patients with suspect cases (influenza A [10 patients], HMPV [4 patients], picornavirus [4 patients], influenza B [1 patient], RSV [1 patient], and PIV3 [1 patient]) and 6 (32%) probable SARS patients (all influenza A [4 patients] and HMPV [2 patients]). Serologic testing identified a likely etiologic agent in specimens from an additional 6 patients who met CDC criteria: 3 (16%) for suspect SARS (influenza A [1 patient], influenza B [1 patient] and adenovirus [1 patient) and 3 (16%) for probable SARS (*M. pneumoniae* [2 patients] and *Chlamydia* spp. [1 patient]). HMPV, whose role in SARS-CoV infection remains undefined, was detected by PCR in four patients with suspect SARS and 2 patients with probable SARS.

**Table 3:** Summary of positive laboratory results at the California Department of Health Services by CDC case criteria^a,b^

Pathogen	Total (N = 165) n (%)	Suspect (N = 51) n (%)	Probable (N = 19) n (%)	Confirmed (N = 2) n (%)	Non-SARS cases (N = 94) n (%)
Influenza A	28 (17)^c^	11 (22)	4 (21)	0	13 (14)
Influenza B	2 (1)	1 (2)	0	0	1 (1)
Respiratory syncytial virus	6 (4)	1 (2)	0	0	5 (5)
Parainfluenza virus types 2–4	6 (5)	1 (2)	0	0	5 (5)
Human metapneumovirus	11 (7)	4 (8)	2 (11)	0	5 (5)
Coronavirus OC43	1 (1)	0	0	0	1 (0)
Coronavirus 229E	0	0	0	0	0
Parainfluenza virus type 1	0	0	0	0	0
Adenovirus	1 (1)	1 (2)	0	0	0
Picornavirus	12 (7)	4 (8)	0	0	8 (9)
Mycoplasma pneumoniae	3 (2)^c^	0	2 (11)	0	1 (1)
Chlamydia spp.	1 (1)	0	1 (5)	0	0
SARS-CoV^d^	2 (1)	0		2 (100)	0
Total positive	73 (44)	23 (45)	9 (47)	2 (100)	39 (42)

^a^Using criteria for suspect, probable, or laboratory-confirmed severe acute respiratory syndrome (SARS) recommended by the Centers for Disease Control and Prevention (CDC). ^b^Includes polymerase chain reaction (PCR), culture, and serologic tests. ^c^One patient had evidence of co-infection with both influenza A (PCR and culture) and *M. pneumoniae* (immunoglobulin [Ig] M detection). ^d^SARS-CoV, SARS-associated coronavirus.

## Discussion

From March to July 2003, California reported more patients who met criteria for suspect and probable SARS than any other state. Many emergency rooms, hospitals, and public health offices were overwhelmed. Hundreds of persons were evaluated by local counties before being reported to CDHS, where they were classified as having suspect or probable SARS. Of these patients, more than one-third had a pathogen detected that was considered a likely cause of their condition based on their clinical features and course of illness. Twenty-one (81%) of these pathogens were identified by RT-PCR within an average of 4 days. In California, determining a commonly recognized cause for an influenzalike illness allowed cases to be removed from the suspect or probable SARS categories. The resultant removal of a SARS designation alleviated the required epidemiologic investigation, hospitalization or isolation, strict infection control precautions, and additional specimen collection and contact tracing.

Although viral or bacterial co-infection with SARS remains possible, only a few SARS-CoV–infected case-patients worldwide have had documented evidence of dual infection, including *C. pneumoniae*, *M. pneumoniae*, and HMPV (*10**–**13*). Most case reports of laboratory-confirmed SARS are noteworthy for the lack of other viral agents (*11**,**14**,**15*). Accordingly, SARS can be ruled out when common culprit viral pathogens are detected in areas without known community transmission of SARS. However, several factors should be considered before discontinuing further evaluation for possible SARS when a likely alternative cause has been identified, including the following: 1) strength of the epidemiologic link to SARS, 2) specificity of the diagnostic testing performed, and 3) consistency of the clinical features and course of illness for the alternative diagnosis. Should SARS become reestablished, these exclusion criteria may need to be reevaluated and applied with particular caution in patients with strong epidemiologic exposure in the context of community transmission.

Applying molecular techniques to outbreak investigations is a relatively recent approach. The limitations of molecular testing include the possibility of false-positive results caused by specimen contamination during processing and false-negative results from primer mismatch or inhibitors in the specimen. Ideally, in the diagnostic setting, positive results by molecular techniques should be confirmed by either testing another specimen by the same method or the same specimen by another method, such as immunoassays or culture. Also, detecting an agent by PCR does not always indicate the true cause of infection; it may instead signify nasopharyngeal carriage or simply be an "innocent bystander."

Nevertheless, given the nonspecific initial signs and symptoms of patients with SARS, the capacity to rapidly diagnose common respiratory infections by using sensitive PCR methods offers advantages in the context of a respiratory outbreak. We found that applying a broad diagnostic molecular panel during the SARS outbreak enabled timely identification of agents of common respiratory viral infections in more than one third of patients with suspect or probable SARS. Although serologic testing aided identification of selected atypical pathogens, the requirement for paired serum specimens did not allow timely removal of a SARS designation. Even when a rapid and definitive diagnostic test for SARS becomes available, laboratories capable of performing molecular-based diagnostic testing, especially for influenza, should be maintained and strengthened.
